# Diversity and feeding strategies of soil microfauna along elevation gradients in Himalayan cold deserts

**DOI:** 10.1371/journal.pone.0187646

**Published:** 2017-11-13

**Authors:** Miloslav Devetter, Ladislav Háněl, Klára Řeháková, Jiří Doležal

**Affiliations:** 1 Institute of Soil Biology, Biology Centre of The Czech Academy of Sciences, České Budějovice, Czech Republic; 2 Centre for Polar Ecology, Faculty of Science, University of South Bohemia, České Budějovice, Czech Republic; 3 Section of Plant Ecology, Institute of Botany of The Czech Academy of Sciences, Třeboň, Czech Republic; 4 Institute of Hydrobiology, Biology Centre of The Czech Academy of Sciences, České Budějovice, Czech Republic; 5 Department of Botany, Faculty of Science, University of South Bohemia, České Budějovice, Czech Republic; Helmholtz Zentrum Munchen Deutsches Forschungszentrum fur Umwelt und Gesundheit, GERMANY

## Abstract

High-elevation cold deserts in Tibet and Himalaya are one of the most extreme environments. One consequence is that the diversity of macrofauna in this environment is often limited, and soil microorganisms have a more influential role in governing key surface and subsurface bioprocesses. High-elevation soil microfauna represent important components of cold ecosystems and dominant consumers of microbial communities. Still little is known about their diversity and distribution on the edge of their reproductive and metabolic abilities. In this study, we disentangle the impact of elevation and soil chemistry on diversity and distribution of rotifers, nematodes and tardigrades and their most frequent feeding strategies (microbial filter-feeders, bacterivores, fungivores, root-fungal feeders, omnivores) along two contrasting altitudinal gradients in Indian NW Himalaya (Zanskar transect from 3805 to 4714 m a.s.l.) and southwestern Tibet (Tso Moriri transect from 4477 to 6176 m a.s.l.), using a combination of multivariate analysis, variation partitioning and generalized additive models. Zanskar transect had higher precipitation, soil moisture, organic matter and available nutrients than dry Tso Moriri transect. In total, 40 species of nematodes, 19 rotifers and 1 tardigrade were discovered. Species richness and total abundance of rotifers and nematodes showed mid-elevation peaks in both investigated transects. The optimum for rotifers was found at higher elevation than for nematodes. Diversity and distribution of soil microfauna was best explained by soil nitrogen, phosphorus and organic matter. More fertile soils hosted more diverse and abundant faunal communities. In Tso Moriri, bacterivores represented 60% of all nematodes, fungivores 35%, root-fungal feeders 1% and omnivores 3%. For Zanskar the respective proportions were 21%, 13%, 56% and 9%. Elevational optima of different feeding strategies occurred in Zanskar in one elevation zone (4400–4500 m), while in Tso Moriri each feeding strategy had their unique optima with fungivores at 5300 m (steppes), bacterivores at 5500 m (alpine grassland), filter-feeders at 5600 m and predators and omnivores above 5700 m (subnival zone). Our results shed light on the diversity of microfauna in the high-elevation cold deserts and disentangle the role of different ecological filters in structuring microfaunal communities in the rapidly-warming Himalayas.

## Introduction

Soil microfauna such as rotifers, nematodes and tardigrades represent important components of cold ecosystems [1, 2, 3, 4] and are dominant consumers of microbial communities [5]. Cold desert soil microfauna have mainly been studied in polar regions [6, 7, 8, 9], and only rarely at lower latitudes [10]. The largest mountain deserts in the Himalayas and Tibet remain unexplored. Here, data on diversity and distribution of rotifers, nematodes and tardigrades and their feeding strategies along two prominent elevational gradients in Indian Himalaya and south-westernmost Tibet are presented.

There is growing interest in belowground diversity as soil biota has a crucial role in ecosystem functioning, especially in organic matter turnover and nutrient mineralization [11, 12, 13, 14, 15]. Understanding of the role of soil biota in ecosystem functioning is complicated by the complex interactions and feedbacks between different soil trophic levels [16]. The high heterogeneity and complexity of the soil physico-chemical environment is an additional complication. Cold ecosystems have characteristics which make it easier to identify relevant environmental drivers and community responses in soil biota. This can lead to the unravelling of the underlying mechanisms responsible for the observed patterns of species distribution and diversity. In particular, species-poor communities are easier to explore through their less complex species interactions, higher vulnerability and susceptibility to climate change, as well as less redundancy in cold desert ecosystems [7, 17]. Such knowledge may potentially help to predict the response of soil biota to climate change in other ecosystems [8].

Organisms inhabiting cold desert ecosystems are generally well-adapted to water shortage and freezing, and can survive extended periods in unfavorable conditions [18, 19]; and can respond immediately to short-term favorable conditions following thawing and snow melt [20, 21]. In the high mountains, such favorable periods are frequently limited to a few days or weeks during summer, when communities are dependent on water from melting snow or accidental precipitation, especially in arid mountains. Here, snow accumulation seems to be a crucial factor determining moisture conditions during the short summer season [22, 23, 24, 25].

Invertebrates belonging to the microfauna undergo anhydrobiosis, which in contrast to other dormant invertebrates, allows them to be active in just a short time after sudden soil wetting at appropriate temperatures [18]. They are very unevenly distributed in relation to vegetation and food sources and this unevenness is more distinctive than in other environments [26].

Soil animal species exhibit a great variation in morphological, physiological, and behavioural traits [27] which may help their response to changes in climate conditions. At high Himalayan altitudes, typical groups of soil fauna such as springtails or oribatides are limited by desert conditions, therefore the list of feeding traits originate mainly from those of nematodes and rotifers. Such groups are also mainly responsible for litter decomposition and soil nutrient dynamics [28, 29]. However, Wolters (2001) [30] proposed that the number of soil animal species needed to maintain ecosystem functioning may depend on the number of functions investigated. To gain further insight into how decomposer species diversity affects ecosystem functioning, the effects of environmental limitations on various feeding strategies were analyzed in our study.

The diversity of vascular plants in mountains is shaped by elevation; plants often have a unimodal, mid-elevation species richness peak. In Ladakh, plants grow from 3000 to 6150 m with diversity culminating between 4500 and 5500 m [31]. Different patterns have been recognized in phototrophic microbes [32], which are the pioneers forming biological soil crusts, together with lichens, mosses and microfungi. Unlike vascular plants, which have a mid-elevation species richness peak, the total biovolume and diversity of phototrophs increased with elevation and soil water availability, especially within biological soil crusts [33]. Microfaunal groups are the only consumers of such biological crusts because higher animals are not able to survive under such severe conditions in reasonable abundance [34]. The question is whether the diversity and abundance of soil microfaunal groups will show a mid-elevation richness peak similar to vascular plants, or will follow the linear trend of increasing richness with elevation as observed in soil phototrophs.

The aims of this study were to determine the key factors driving variation in soil microfaunal community composition and diversity along an elevational gradient together with the most important feeding strategies of soil microfauna. The diversity and compositional changes were compared to changes in soil nutrient concentration and water availability. Two elevational transects, firstly in the wetter Himalayan Range and secondly in drier Tibetan Plateau were compared.

## Methods

### Study area

The research was conducted in Ladakh, Jammu and Kashmir States, India. All necessary permits were obtained for the field studies described below from the University of Kashmir, Srinagar, and local authorities of Leh District. Due to its position in the rain shadow north of the Great Himalaya Range, the Ladakh region is arid and receives very little precipitation (< 150 mm), especially at the foothills between 3000–4500 m [35], where evaporation exceeds precipitation [36]. However, the Zanskar area is more humid than the Tibetan Plateau where at elevations above 4500 m, precipitation tends to increase [37].

Field samples were taken in August 2010 in two areas of Ladakh (S1 Fig): 1) The Himalayan transect from Darcha over Singola Pass to Zanskar valley (from about 3800 to 4700 m elevation, N32° 45’ E77° 08’ to N32° 58’ E77° 15’), and 2) The Tibetan transect from Tso Moriri Lake to Shukule Peak in Chamser Kangri Massif (from about 4700 to 6200 m, N32° 58’ E78° 21’ to N32° 59’ E78° 28’). The bedrock in the Himalayan transect consists of siliceous granite and sedimentary rocks, while that in Tibet consists of gneisses. These two transects cover elevation ranges from 3805 m in Darcha to 6176 m at Shukule Peak. The vegetation zonation included semi-deserts and steppes at lower elevations (collectively referred to as steppes hereafter), and alpine meadows, screes and the subnival zone at higher elevation. Screes represent a habitat that partly overlaps in elevation range with alpine meadows and the subnival zone. The cover of vascular plant vegetation ranged from 0 to 50%. The data on vegetation type and cover at the same sampling points were well documented [31, 38, 39].

### Data collection

The sampling points located along an elevational transects were 200–400 m from each other to cover all major vegetation types. Each transect consisted of five sampling sites with 3 replicate plots, situated up to one hundred meters from each other and independent soil samples were taken at each plot. These samples consisted of four cores at Tso Moriri and five cores at Zanskar and were taken randomly with a soil auger (area 25 cm^2^, depth 4 cm). We collected the soil samples during the first half of August, which is the most favourable period, in terms of temperature and soil water availability, for development of soil faunal communities and hence our data are representative of the study area. Given a short growing period, which can be only about 4 weeks at 6000 m, the field data collection took place when night temperatures stayed above zero.

The volumes of the 4–5 randomly-taken soil samples were pooled into one composite sample and weighed. Wet samples were air-dried in the field and weighed again to measure the gravimetric soil moisture content. This procedure does not disrupt the samples because of anhydrobiosis ability of animals and frequent natural drying in the field. Later in the laboratory, three replicate 15–20 g dry mass sample from each plot were moisturized for 24 hours and extracted using L-C extraction [40]. This behavioural method for living specimens, based on their own activity, is suitable and quantitative method commonly used for all groups of microfauna [40], avoiding unfavourable conditions such as heat and light. In contrast to other methods, it does not discriminate any microfaunal groups. Rotifers, nematodes and tardigrades were enumerated by direct counting under an inverted microscope (Leica DMIL) with 25x total magnification; detailed determination was carried out on slides. Dry samples were processed up to 3 months from sampling. Rotifers were determined using Donner (1965) [41] in light of later descriptions. As a basis for the determination of nematodes, the most recent monographs were used [42, 43, 44]. Because Andrássy provided keys to European fauna, the determination of nematodes was also carried out using other studies mostly cited in his books or newer studies such as monograph on Tylenchidae [45]. Nevertheless, many well-distinguishable species could not be identified with species described in available taxonomic papers. These may be new to science or may represent local populations with morphometrical variation outside the limits of species populations described from other parts of the world. One species of tardigrade is new for science and its description is being prepared.

Eight feeding groups in the soil microfauna were distinguished: microbial scraper rotifers, microbial filter-feeding rotifers, bacterivorous nematodes, fungivorous nematodes, root-fungal feeding nematodes, omnivorous nematodes, predacious nematodes and omnivorous tardigrades (S1 Table). Allocation of nematodes to trophic groups was carried out using Yeates et al 1993 [5], with modifications according to Háněl 2010 [46].

### Physico-chemical characteristics of soil

The following parameters were measured in all soil samples: total nitrogen (TN), N-NH_4_^+^, N-NO_3_^-^, P-PO_4_^3-^, Ca^2+^, Mg^2+^, Na^+^, K^+^, pH, soil moisture and organic matter (OM). Soil physico-chemical analyses were conducted in accordance with the standardised methods of the Association of German Agricultural Analytical and Research Institutes [47]. Soil pH was potentiometrically measured in a suspension with 0.01 M CaCl_2_. Plant available N and P contents of the soil samples were analysed colorimetrically with an FIAstar 5010 Analyzer (Foss Tecator AB, Höganäs, Sweden) and the cations with AAS (SpectrAA 640, Varian Techtron, Melbourne, Australia).

### Statistical analyses

In order to assess the effects of mountain range, elevation, and soil physico-chemical parameters on species compositional changes in microfaunal assemblages, we performed a Detrended and Canonical Correspondence Analyses (DCA and CCA) [48]. To separate the net effect of individual variables on species composition of the soil microfauna, we performed a variation partitioning for three groups of environment predictors [48]. Prior to the variation partitioning analysis, separate CCA was run on soil physico-chemical characteristics to select those with significant impact on microfaunal assemblages using interactive forward selection and Monte-Carlo randomization tests (999 permutations). Variation partitioning was based on adjusted explained variation (corresponding to R^2^_adj_ measure used in linear regression) and conditional (unique) effects of individual groups of predictors were tested. During stepwise selection of explanatory variables, estimated Type I errors were adjusted by transforming them into FDR (false discovery rates). Data were log-transformed prior analysis. The results of multivariate analyses were visualized as a biplot ordination diagrams with the species labelled by the first three letters of the generic and first three letters of the species names. Redundancy analysis (RDA) was used to test the effect of elevation on variation in soil physico-chemical properties [48, 49].

We further modelled elevational changes in richness and abundance of soil microfauna and their trophic groups and soil chemical properties using response curves fitted by generalized additive models, (GAM) assuming Gaussian, Binomial or quasi-Poisson distribution, depending on the nature of a particular response variable, and stepwise selection of alternative models using the Akaike Information Criterion. The GAM framework is useful for discovering elevation-specific patterns, does not rely on any predefined relationships, and provides easily interpretable visualizations [49]. The ordination and response analyses were performed in Canoco 5 [48].

Conditional inference trees [a type of classification and regression tree (CART)] were used to find a parsimonious subset of environmental predictors of soil microfaunal richness and abundance using R package party [50]. The method belongs to non-parametric regressions, making a dichotomous tree, which can be used as a predictive model to get insights into which environmental factors contribute to high/low values of soil variables. This type of classification and regression tree has several crucial advantages over other approaches (e.g. traditional CART algorithm), including (1) the statistical testing of each split through permutation, (2) no need for problematic pruning of over-fitted trees, and (3) no selection bias towards variables with many possible splits or missing values [50].

## Results

### Environmental soil variables

**Fig 1:**
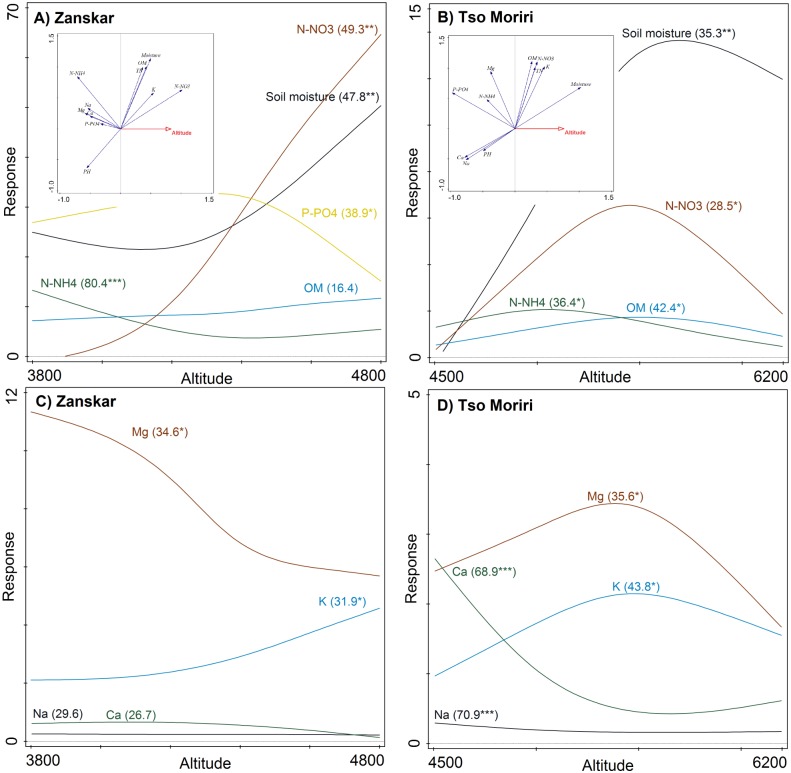
Altitudinal changes in soil physico-chemical properties in Zanskar and Tso Moriri. Generalized Additive Models were used to analyse altitudinal responses. Redundancy analysis biplots show interrelationships between soil variables and elevation. For units, see Table 1. Explained variation (R^2^) and estimated Type I errors (P values *<0.05, **<0.01, ***<0.001) are shown in parentheses.

Zanskar had significantly higher organic matter, nitrogen (all forms) and soil water content (Table 1). Tso Moriri, on the other hand, had a significantly higher concentration of calcium and pH. Significant elevational changes were found in most soil variables (Fig 1). In Zanskar, nitrate, soil moisture and potassium increased significantly with elevation, while magnesium, ammonium and sodium decreased with elevation, and phosphorus and pH had a hump-shaped pattern. In Zanskar, organic matter was positively related with soil total nitrogen (r = 0.87), nitrates (r = 0.75), and soil moisture (r = 0.74). Soil reaction (pH) was positively related to phosphorus (r = 0.63) and negatively to organic matter content (r = -0.70) and soil moisture (r = -0.65).

**Table 1:** Soil physico-chemical properties of Zanskar and Tso Moriri transects in Indian NW Himalaya. Mean, minimum and maximum values are shown together with *F*-test and estimated Type I errors (P values *<0.05, **<0.01, ***<0.001) for differences between two transects.

	Zanskar	Tso Moriri	F
**Altitude (m asl)**	4331 (3805–4714)	5339 (4477–6176)	37.6***
**Soil Moisture (%)**	30.4 (12.9–56.8)	9.1 (0–16.8)	24.9***
**Organic Matter (%)**	9.1 (4–20.2)	1.2 (0.4–3.4)	58.1***
**pH**	5.7 (3.6–7.8)	7.3 (6.1–8.6)	20.3***
**N-NH_4_ (mg kg^-1^)**	6.2 (1.9–13.6)	1.3 (0.5–5.4)	16.6***
**N-NO_3_ (mg kg^-1^)**	27.7 (1.6–106.4)	3.7 (0.3–26.6)	8.26**
**Total N (mg kg^-1^)**	4068 (1427–4585)	542 (160–1587)	22.4***
**P-PO_4_ (mg kg^-1^)**	27.9 (10.9–46.9)	24.9 (2.7–49.7)	ns
**Na (g kg^-1^)**	0.23 (0.2–0.3)	0.19 (0.1–0.4)	5.1*
**K (g kg^-1^)**	3 (1.3–6.1)	1.7 (0.7–4.1)	9.9**
**Ca (g kg^-1^)**	0.5 (0–1.1)	1.1 (0.3–4.4)	ns
**Mg (g kg^-1^)**	7.8 (1.5–13.5)	2.7 (0.1–7.2)	21.7***

In Tso Moriri, most variables had a hump-shaped relationship with elevation. Soil moisture, organic matter, total N, nitrate, magnesium and potassium peaked at 5400–5800 m elevation, while phosphorus and ammonium at around 5000 m; calcium and sodium showed valley-shaped relationships, with the lowest values at mid-elevations and the highest values at the lowest elevations (Fig 1). RDA showed that elevation explained 48.5% and 33.9% variability in soil physico-chemical properties in Zanskar and Tso Moriri (both *P* < 0.01), respectively.

### Environmental determinants of soil microfaunal assemblages

In total, 40 species of nematodes, 19 species of rotifers and 1 tardigrade species were discovered (S1 Table). Animals were detected in all samples from both transects. The nematodes made up the most abundant part of the community, both in abundance of individuals as well as species diversity. The species richness differed between two studied mountain ranges (Table 2). More nematode species were found in Tso Moriri (32) than in Zanskar (22) while more rotifer species occurred in Zanskar (15) than in Tso Moriri (10).

**Table 2:** Summary table of abundance and species richness of soil microfauna in two elevational gradients in NW Himalayas. Abundance x 1000 ind m^-2^, mean (SD).

Zanskar elevation sites	3800		4200		4400		4550		4700	
Rotifera species richness	1		3		6		11		2	
Rotifera abundance	1.7	(2.3)	3.9	(2.2)	13.3	(14.6)	18.0	(5.8)	4.0	(5.7)
Nematode species richness	9		6		17		10		4	
Nematode abundance	12.5	(4.7)	19.7	(10.4)	81.1	(37.5)	64.2	(9.1)	12.8	(3.5)
Tso Moriri elevation sites	4500		5000		5400		5800		6200	
Rotifera species richness	0		2		5		5		2	
Rotifera abundance	0		1.1	(0.8)	15.2	(11.1)	3.6	(3.1)	1.1	(0.8)
Nematode species richness	4		11		18		17		2	
Nematode abundance	3.6	(0.7)	84.5	(85.1)	413.6	(32.4)	30.8	(10.2)	5.2	(2.6)
Tardigrada abundance	0		0		0		0		1.5	(1.2)

**Fig 2:**
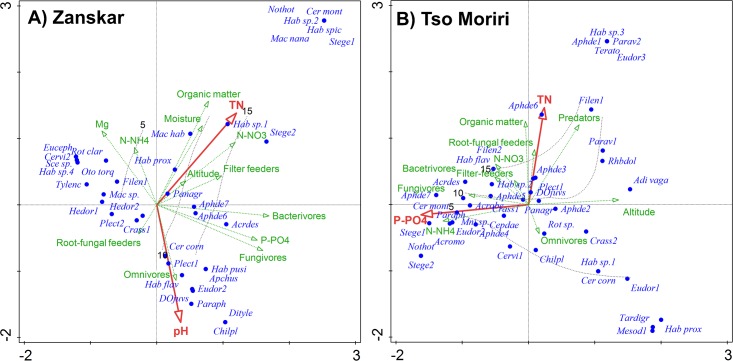
The CCA ordination of soil microfauna. The most explaining environmental variables were determined by forward selection (red arrows) for (A) Zanskar and (B) Tso Moriri mountain ranges. Source data are log-transformed, species are represented by blue dots (for full names, see S1 Table). Feeding groups, altitude and other environmental variables are passively projected into the CCA ordination diagram (green arrows). The isolines of species richness of samples (grey lines) are fitted using the *Loess* smoothing splines.

The CCA analysis showed that both elevation gradients significantly differed in microfaunal composition as well as in the response to environmental conditions (Table 3, Fig 2). There were two well-distinct groups of microfauna samples taken either in Tso Moriri or Zanskar, with the Tso Moriri transect showing greater between-sample variability then the Zanskar transect. The length of the gradient of the first DCA ordination axis was 7.8 SD, which indicates high species turnover and prevailing unimodal elevation responses in animal assemblages.

**Table 3:** Explained microfaunal compositional variation by individual environmental variables. The CCA multivariate analyses and permutation test were applied for Zanskar and Tso Moriri faunal assemblages (both nematoda and rotifera) in Indian NW Himalayas. Explained variation (*R*^*2*^) and estimated Type I errors (*P* values *<0.05, **<0.01, ***<0.001) are shown. Best predictors selected by CCA forward selection models are depicted in bold.

	Zanskar			Tso Moriri		
	All	Nematoda	Rotifera	All	Nematoda	Rotifera
**Altitude**	ns	ns	ns	13.4***	13.8***	18.5*
**Soil Moisture (%)**	ns	ns	ns	10.5*	ns	ns
**Organic Matter (%)**	13.0*	10.6*	ns	10.7*	ns	ns
**pH**	11.5*	12.8*	ns	ns	ns	ns
**N-NH_4_ (mg kg^-1^)**	ns	10.1*	ns	10.8*	12.6*	ns
**N-NO_3_ (mg kg^-1^)**	9.9*	ns	16.6a	10.3*	ns	ns
**Total N (mg kg^-1^)**	14.0*	13.6*	ns	11.6*	11.7*	ns
**P-PO_4_ (mg kg^-1^)**	10.6*	21.4**	ns	16.3***	16.9***	19.6*
**Na (g kg^-1^)**	ns	ns	ns	ns	ns	ns
**K (g kg^-1^)**	ns	ns	ns	ns	ns	ns
**Ca (g kg^-1^)**	ns	ns	ns	ns	ns	ns
**Mg (g kg^-1^)**	10.2*	11.0*	ns	10.2*	ns	ns

The combined effect of altitude, mountain range, and selected soil chemical properties explained 27.8% of the total microfaunal compositional variation and was highly significant (*F* = 3.0, *P* = 0.001). Concerning marginal effects of each explanatory variable (analyses with no covariables), CCA showed that the contribution to compositional variation was 9.3% for mountain range (*F* = 2.9, *P* = 0.001), 8.8% for altitude (*F* = 2.7, *P* = 0.001), and 20.1% (*F* = 3.2, *P* = 0.001) for soil chemical predictors. CCA stepwise selection of a parsimonious subset of soil predictors included phosphorus (8.2%), organic matter (6.2%), and total nitrogen (5.7%). Variation partitioning confirmed that microfaunal assemblages are most related to the measured soil chemistry and less to the elevation and mountain range. Of the total explained variation, the unique effect of elevation explained 0.5%, 0.6% was explained uniquely by mountain range and 3.6% by soil chemistry (the last group had significant effects at *P*<0.05). This does not mean that first two predictors were ineffective, their independent effects were significant.

The separate multivariate analyses revealed that phosphorus, organic matter, magnesium, total nitrogen and pH were significant predictors of nematode compositional variation in Zanskar (Table 3), while phosphorus, total nitrogen, ammonium and elevation in Tso Moriri. The variability explained by organic matter represents a difference between two studied transects, and is highly correlated with total nitrogen and elevation, with the Tso Moriri transect having less organic matter, soil N and higher pH. The best soil predictors of compositional variation in rotifers included nitrates in Zanskar and phosphorus and elevation in Tso Moriri.

The CCA analysis for Zanskar (Fig 2A and Table 3) shows that the plots with higher nitrates, organic matter and soil moisture are associated with higher faunal diversity, filter feeders and taxa such as *Macrotrachela nana*, *Stegelletina similis*, *Macrotrachela habita* and *Habrotrocha spicula*. Among species associated with lower phosphorus and higher magnesium and ammonium soils were *Eucephalobus oxyuroides*, *Otostephanos torquatus* and *Tylenchus naranensis*. The higher pH soils host higher diversity and species such as *Habrotrocha pusila*, *H*. *flaviformis* and *Dorylaimida juveniles*. The higher phosphorus concentration is associated with higher abundances of bacterivores and fungivores represented by taxa such as *Acrobeloides tricornis* and *Aphelenchoides* spp.

The CCA analysis for Tso Moriri shows that the first ordination axis is most related to phosphorus, which decreases with increasing elevation (Fig 2B). The lower elevation plots with the higher phosphorus content are associated with lower diversity, higher proportion of fungivores and species such as *Stegelletina devimucronata*, *Acrobeloides tricornis* and *Paraphelenchus pseudoparietinus*. The higher elevation plots with lower phosphorus content are associated with taxa such as *Adineta vaga* and *Rhabdolaimus terrestris*. The second axis is most related to total nitrogen and organic matter, with the highest values at mid elevations, which host higher diversity, total vegetation cover, higher proportion of predators and root fungal feeders and taxa such as *Filenchus butteus*, *F*. *quartus* and *Aphelenchoides* spp.

### Altitudinal changes in species richness and abundance of soil microfauna

**Fig 3:**
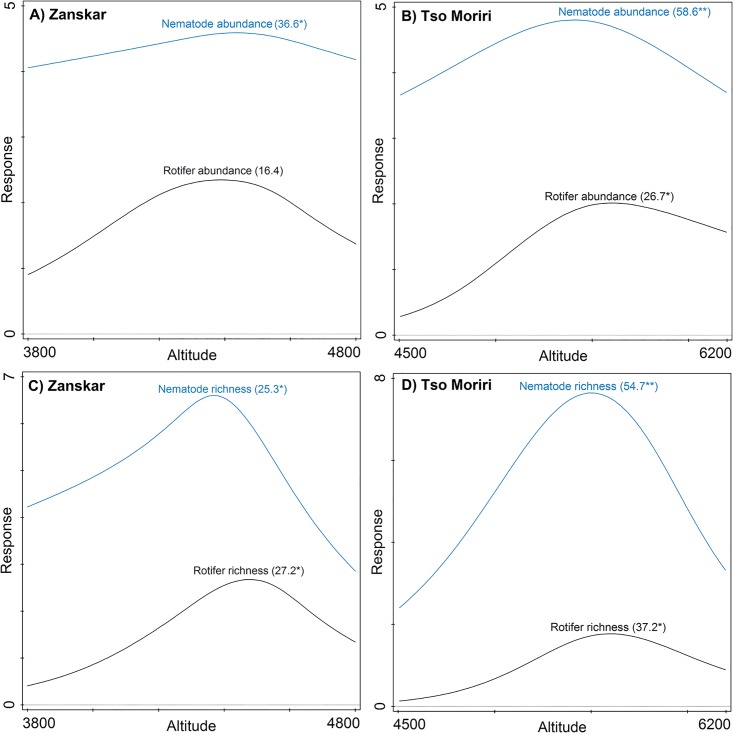
Altitudinal changes in abundance and richness of rotifers and nematodes along two gradients (Zanskar and Tso Moriri) in Indian NW Himalaya. Generalized Additive Models were used to analyse altitudinal responses of soil microfauna. Abundances were log-transformed. Explained variation (R^2^) and estimated Type I errors (P values *<0.05, **<0.01, ***<0.001) are shown in parenthesis.

Total richness and abundance of rotifers and nematodes showed a significant unimodal response in Tso Moriri with a mid-elevation peak, while in Zanskar only nematodes exhibited a marginally significant unimodal abundance response (Fig 3). In Tso Moriri, the optimum for rotifers was found at higher elevation than for nematodes. In Tso Moriri, nematode and rotifer species richness and abundance was significantly and positively related to soil moisture, organic matter, nitrates, total N and magnesium, and negatively related to soil pH and sodium. In Zanskar, nematode species richness and abundance was positively related to soil phosphorus, while the rotifer species richness and abundance in Zanskar was significantly positively related to total soil nitrogen and nitrates (Table 4).

**Table 4:** Relationship between soil properties and richness and abundance of microfaunal communities. Explained variation (R^2^) and estimated Type I errors (P values *<0.05, **<0.01, ***<0.001) from generalized linear model are shown. A parsimonious subset of environmental predictors selected by conditional inference trees are in bold.

	Zanskar				Tso Moriri			
	Nematoda		Rotifera		Nematoda		Rotifera	
	Richness	Abundance	Richness	Abundance	Richness	Abundance	Richness	Abundance
**Soil Moisture**	ns	ns	ns	0.58*	0.50*	0.58*	0.81***	0.61*
**Organic Matter**	ns	ns	0.55*	Ns	0.79***	0.80***	0.69**	0.51*
**pH**	0.63*	ns	ns	Ns	-0.60**	-0.45*	ns	ns
**N-NH4**	ns	-0.51*	ns	Ns	ns	ns	ns	ns
**N-NO_3_**	ns	Ns	0.61*	0.80**	0.55*	0.70**	0.75**	0.53*
**Total N**	ns	Ns	0.68**	0.55*	0.83***	0.62*	ns	ns
**P-PO_4_**	0.77***	0.68**	0.49*	0.44*	ns	ns	ns	ns
**Na**	ns	ns	ns	Ns	ns	ns	ns	-0.45*
**K**	ns	ns	ns	Ns	0.59*	0.77***	0.73**	0.54*
**Ca**	ns	ns	ns	Ns	ns	ns	ns	ns
**Mg**	ns	ns	ns	Ns	0.53**	0.74**	0.64**	ns

### Feeding strategies and their environmental determinants

**Fig 4:**
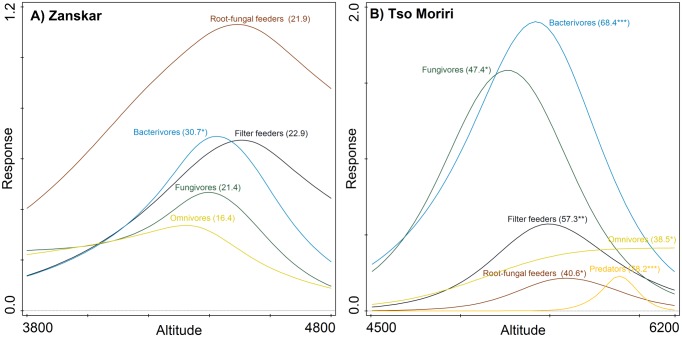
Altitudinal changes in abundance of microfaunal feeding groups along two mountain transects (Zanskar and Tso Moriri) in Indian NW Himalayas. Generalized Additive Models were used to analyse altitudinal responses of omnivorous nematodes (Omnivores), filter-feeding rotifers (Filter-feeders), fungivorous nematodes (Fungivores), bacterivorous nematodes (Bacterivores), predacious nematodes (Predators) and root-fungal feeding nematodes (Root-fungal feeders). Explained variation (R^2^) and estimated Type I errors (P values *<0.05, **<0.01, ***<0.001) are shown in parenthesis.

**Fig 5:**
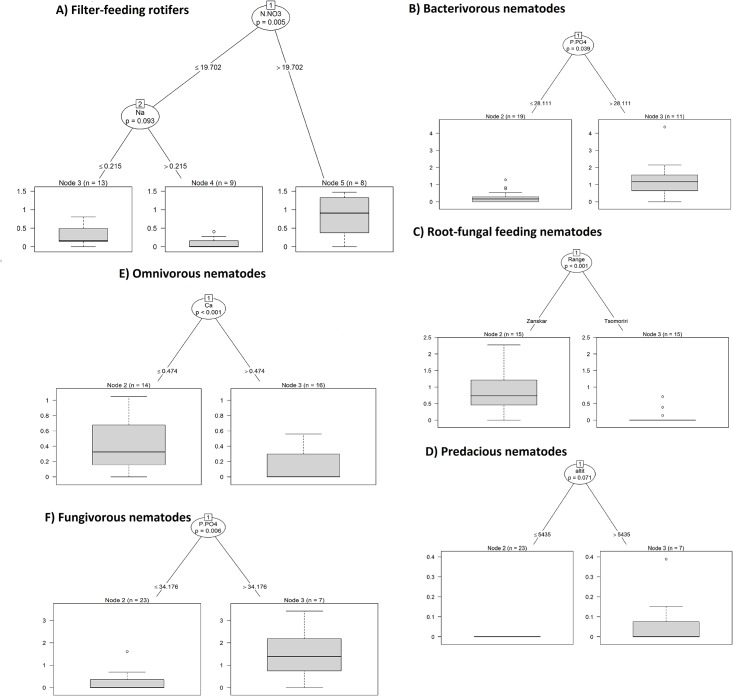
Conditional inference trees showing a significant effect of environmental factors on abundance of individual microfaunal feeding groups. In each split of the tree, all predictors are tested and the one that best discriminates between higher and lower values is selected. Each split of the tree is described by the factors associated with the split (ovals), the permutation-based significance of the split (*P*-value) (ovals) and the level at which the split occurs (line between ovals and boxes). The Box-and-Whisker plot and number of plots (*n*) is given at each terminal node.

Metazoan soil microfauna exhibited various feeding strategies. The two regions substantially differed in the proportions of trophic groups in nematode assemblages. In Tso Moriri, bacterivores represented almost 60% of all nematodes, fungivores 35%, omnivores 3% and root-fungal feeders 1%. For Zanskar the respective proportions were 21%, 13%, 9% and 56%. The high proportion of microbivores in Tso Moriri reflects high population densities of bacterivorous *Panagrolaimus* cf. *rigidus* and of fungivorous *Aphelenchoides* sp.4. On the other hand, root-fungal feeding *Filenchus quartus* occurred in all Zanskar samples except one. In Tso Moriri, root-fungal feeding *Filenchus* occurred only in three of the fifteen samples. Plant parasites of the order Tylenchida were absent from all sites surveyed and predacious *Paravulvus* occurred very rarely at Tso Moriri only (<1%). In rotifers, microbial filter feeding is the dominant strategy and scraping is negligible. However, this strategy is very common in Zanskar, where its presence is not significantly dependent on elevation as in Tso Moriri. Here, the unimodal quadratic model suggested a significant peak at 5500 m and rotifers were abundant in all sites (Fig 4). Bacterivory in nematodes is a frequent feeding strategy in both transects. In both cases, the GAM model showed very clear and significant peaks at 4450 m in Zanskar and at 5500 m in Tso Moriri (Fig 4). Also, in fungivorous nematodes, there was significant peak at 5300 m in Tso Moriri and marginally significant at 4400 m in Zanskar. The abundance of root-fungal feeders was higher in Zanskar than in Tso Moriri, with elevation optima at 4500 and 5600 m, respectively. The omnivorous nematodes had a tendency to peak at 4350 m elevation in Zanskar, while in Tso Moriri their abundance increased significantly with elevation. The conditional inference tree analyses showed that each feeding group is driven by different environmental drivers (Fig 5). The filter-feeding rotifers have higher abundance at higher nitrate and lower sodium levels. Fungivorous and bacterivorous nematodes achieve significantly higher abundance at higher phosphorus concentration. Root-fungal feeding nematodes have significantly higher abundance in Zanskar and predacious nematodes at high elevation above 5400 m in Tso Moriri.

## Discussion

### Abundance and taxonomic richness

Communities of hydrobionts are well developed in Ladakh cold deserts, with animals capable of surviving under severe conditions of water scarcity and freezing temperatures. The abundances of rotifers and nematodes recorded in Ladakh are within the ranges reported from temperate [46, 51, 52, 53, 54, 55] and polar latitudes [26, 34, 56, 57], however they are rather close to the lower values of population abundance. Tardigrades, on the other hand, are very rare in Ladakh soils. Nematodes dominated in the studied samples, which is also common in the other types of environment [58, 59, 60, 61] but exceptions to this rule exist [34].

Relatively low nematode richness at individual sites (1–12 species) is very likely caused by harsh climates that can limit the variety of nematodes in polar and arctic latitudes [62]. Velasco-Castrillón et al. 2014 reported 68 nematode species for Antarctica [63]. The similar number can be found in a single agricultural field in Central Europe [64]. But a total of 40 species found in the Zanskar and Tso Moriri transects in NE Himalayas is a relatively high number. For instance, Loof (1971) surveyed 70 samples in Svalbard where the number of nematode species varied from 3 to 24 and the total number of species was about one hundred because some species recorded probably comprised several species [65].

Nevertheless, it is interesting that more species of nematodes were distinguished (32) in Tso Moriri with nutrient-poor soils than in Zanskar (22) with nutrient-rich soils. This may be due to a greater number of microhabitat patches in Tso Moriri, which stretches across a longer altitudinal gradient. Rotifers had more species in Zanskar (15) than in Tso Moriri (10), therefore competition for (micro)habitat within the two groups of soil microfauna cannot be excluded. The two transects differed mainly in precipitation and soil moisture content. Other variables such as vegetation, nutrient and organic matter contents are related to available soil moisture. Consequently, Zanskar valleys are relatively intensively used for pasture, Tso Moriri is grazed extensively, mainly at lower elevations. A close relationship is expected between the activity of vertebrate herbivores and nutrient deposition—arid climates and short seasons restrict mineralization of organic matter and excrement in the soil. Livestock grazing can affect nematodes of particular feeding traits as discussed below.

### Relationships between abiotic factors, soil microfauna and other biota

The communities of soil microfauna are determined mostly by nutrients and organic matter, which change predictably with elevation. The elevation gradients are steep in both mountain ranges studied with rapidly changing conditions from dry, desert-like sandy plots to a relatively moist subnival zone with compact cushion plants and soil crusts. Altitude and its associated climatic conditions were correlated with nutrient dynamics, so while the nutrient effects were statistically dominant, the effect of altitude cannot be ruled out. In the cold deserts of Antarctica, organic carbon and moisture was also found to be closely related to microfauna, as well as tightly correlated with NP nutrients. Any correlation should be the result of a connection between suitable soil geochemical conditions with soil productivity levels and microbial activity [9]. In our study, soil phosphorus explained greatest proportion of species compositional variation.

This study proved the significant elevational changes in soil microfauna abundance with a prevailing hump-shaped pattern. Changes in soil animal communities, including nematodes, along an elevational gradient have been also found [66, 67]. For microfauna, differences among sites are due to conditions which allow animals to establish populations. However, the populations are not primarily limited by dispersal ability [54]. Soil microfauna can be well dispersed by wind, which is supposedly facilitated when the nematodes, rotifers and tardigrades are in anhydrobiosis [68]. Wind is an important abiotic factor responsible for the dispersion of soil microfauna in terrestrial ecosystems; dispersion in studied cold deserts as well as in alpine and subnival zones is further facilitated by sparse vegetation [69, 70].

The presence and abundance of microfauna is also connected with vascular plant richness and productivity as well as with microbial phototrophic communities (algae, cyanobacteria) and, in particular, biological soil crusts [34]. Altitudinal patterns in abundance and richness of soil fauna follow in general that of vascular plants whose richness and cover also peaked at mid-elevations [31]. It has been reported by Řeháková et al. [32] that phototrophs and bacteria have no elevational limits if liquid water is present for even a short time per year. It is assumed here that this holds for microfauna, even the soils at highest elevations (6175 m) host unique species of tardigrade (in prep.) and some rotifers which are not present at lower elevations, despite more favorable conditions.

### Feeding traits in high-elevation ecosystem

Soil fauna is thought to be composed of generalists in terms of feeding behaviour and competition for similar resources [71], and hence competitive interactions in soil can be less important [15]. However, the different elevational optimum for bacterial feeders, fungivores and other strategies under the severe conditions of the Tso Moriri transect can indicate competition between components of the food web for nutrients and food in soil.

The importance of trophic traits is, however, not only significantly dependent on the elevation in cases where the chemical factors are changing. This suggests that the relative importance of feeding strategies depends on physical factors related to thawing and the vertical distance of the snow line. Interestingly, the peaks of bacterivorous microfauna are at about 800 vertical meters below the snowline in both Zanskar and Tso Moriri, although in the wetter Himalayan Zanskar transect the snowline is about 900 m lower than in the drier Tso Moriri. However, the positive effect of snow fields in soil microfauna is known [25] and it seems that such peaks are developed with a time lag after snow retreat.

The second important point is that peaks of different feeding strategies occur in Zanskar at similar elevations (except for less important omnivores), while in Tso Moriri each of the individual feeding traits have their unique, most preferred elevation. This is likely connected with gradient of soil moisture availability in Tso Moriri, with water stress decreasing from lower-elevation deserts to steppes, and alpine grasslands and then increasing again towards the subnival zone where water starts to be unavailable due to frost. No such severe water gradient exists in Zanskar.

The whole territory of Tso Moriri was marked with the absence of mostly plant parasitic Tylenchida. This nematode group of various feeding and life-history traits is often dominant in natural and man-established ecosystems [5, 72]. Verschoor et al. [73] suggested, that in the temperate climate of Europe, plant-feeding nematodes such as *Heterodera*, *Subanguina*, *Helicotylenchus*, *Geocenamus*, *Tylenchorhynchus* and *Paratylenchus* have annual population cycles while taxa such as Tylenchidae species can produce more generations per year. Cold climates probably hampered the establishment of populations of the true plant parasites while in lower Zanskar Tylenchidae were relatively abundant.

Bacteria-feeding nematodes were positively related to total soluble nitrogen in both Tso Moriri and Zanskar, suggesting that nitrification-denitrification processes may play a crucial role in the below-ground soil interaction—indicators of N availability [74, 75, 76, 77]. Bacteria-feeding nematodes had abundance peak at around 5500–5700 m, which was the elevation belt where Janatková et al. (2003) [33] found the highest values for nitrogenase activity (nitrogen fixation) by soil phototrophs. Nitrogen can be limiting for microbial communities and consequently for microbial-feeding microfauna. Ettema et al. 1999 [76] found population increase in bacterivores with *r*-selected life strategies after N-addition but not fungivores in riparian soil with surplus of water. Sánchez-Moreno and Ferris (2007) found a positive correlation between bacterivore and fungivore abundance with NO_3_^-^ but a negative correlation with NH_4_^+^ in a microcosms experiment [78]. Ou et al. (2005) found a positive correlation between total N and alkali N and total nematode numbers together with bacterivores, fungivores, plant parasites and omnivore-predators [79]. Because nematodes generally play an important role in nitrogen mineralization in a wide variety of soils, and bacterivores are usually more active than fungivores [75, 80], there is no reason why they should not provide this service to soils in the high mountain systems of the Himalayas. This can also explain why Tso Moriri had less nitrogen in the soil—abundant populations of *Panagrolaimus* and *Aphelenchoides* (*r*-selected nematodes) could strongly contribute to the loss of this nutrient from ecosystems where vegetation is too sparse to assimilate the released nitrogen back into the system. In more complex ecosystems in Zanskar with abundant alpine vegetation and a prevalence of root-fungal feeding *Filenchus*, such a loss of nutrients from the soil subsystem was, very likely, reduced in comparison with the less developed Tso Moriri soils. The increase of the abundance and dominance of *Filenchus* in naturally developing grassland was documented by Háněl 2003 [64] and in high mountains forests by Zhang et al, (2015) [81] and can be a good indicator of a structured soil food web [46].

The abundance and diversity of microbial filter feeders as well as bacterivorous nematodes and fungivores demonstrates a significant unimodal elevational response, which can be related to similar types of response as referred to by Degens et al. 2001, who explained unimodal patterns of soil microbial diversity by gradients of stress and disturbance (freezing-thawing cycles frequency) [82]. Contrary to this, other authors did not detect a unimodal response of studied soil groups on disturbance and nitrogen availability in their studies [15, 83].

The processes of symbiosis, herbivory and decomposition, carried out by soil biota, influence plant productivity and the soil habitat as well as hydrological and biogeochemical cycling [84]. In terms of succession pathway, the habitats in higher altitudes of Tso Moriri are in a state of young successional stage with inefficiently linked food webs and nutrient cycling [14]. Climate change could provide higher temperatures and more precipitation favorable for many organisms, thus increasing nutrient retention in the soil of Tso Moriri and intervention of plant parasitic nematodes into the ecosystems of Zanskar. At the very least, the species composition of soil microfauna assemblages may indicate such development. Climate warming is often taken as a negative phenomenon. However, on surveying nematode fauna, Nielsen et al. (2011) [8] came to the conclusion that predicted climate changes are likely to exert a strong influence on nematode communities throughout Antarctica and will generally lead to increasing abundance, species richness and food web complexity, although the opposite may occur locally. Some species adapted to cold, dry environments can diminish or become extinct such as *Scottnema lindsayae* Timm, 1971 in Antarctica [85] and *Acromoldavicus* cf. *mojavicus* in Tso Moriri.

In conclusion, it was found that the soils from both vertical transects differed significantly in their physical and geochemical characteristics and in their microfauna communities. These communities also regularly change with elevation, especially in the nutrient poor and dry Tso-Moriri sites. This study also showed that abiotic variables are correlated with the composition of soil metazoan microfauna. The communities are determined, mainly by nutrient and organic matter contents, which are mostly related to elevation. The most important feeding trait, dominant in the highest elevations of Tso Moriri, is filter feeding (rotiferan) and nematode bacterivory followed by fungivory at lower elevations.

## Supporting information

S1 Fig Map of Ladakh area in Indian NW Himalayas with sampling sites. (DOCX)

S1 Table The species of microfauna of Ladakh soil. Frequency in each elevational transect. Traits: scraper (S), microbial filtrator (MF), bacterivore (B), fungivore (F), root-fungal feeder (RFF), predator (P), and omnivore (O). Codes of species refer to those in ordination diagram.
(DOCX)

S2 Table The source data for analyses. (PDF)
