# Supplementary figures and images for: Diversity and feeding strategies of soil microfauna along elevation gradients in Himalayan cold deserts
Source: PLoS One. 2017 Nov 13;12(11):e0187646. doi: 10.1371/journal.pone.0187646 (PMC5683576; doi:10.1371/journal.pone.0187646)

**S1 Fig Map of Ladakh area in Indian NW Himalayas with sampling sites.**


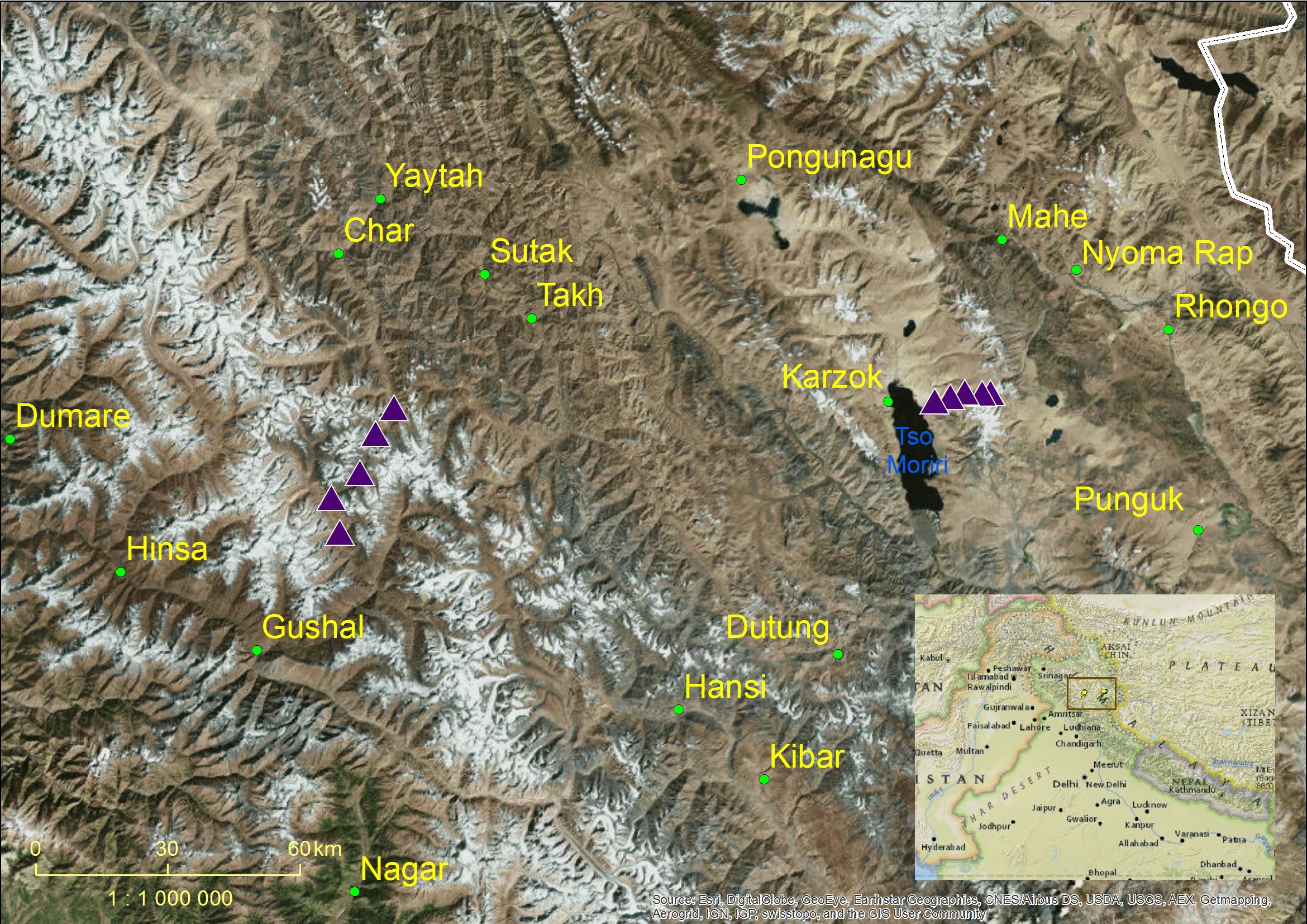

Supplement: S1 Fig — (DOCX) [file pone.0187646.s001.docx]
