# Supplementary material for: Diversity and feeding strategies of soil microfauna along elevation gradients in Himalayan cold deserts
Source: PLoS One. 2017 Nov 13;12(11):e0187646. doi: 10.1371/journal.pone.0187646 (PMC5683576; doi:10.1371/journal.pone.0187646)
Supplement: S1 Table — Frequency in each elevational transect. Traits: scraper (S), microbial filtrator (MF), bacterivore (B), fungivore (F), root-fungal feeder (RFF), predator (P), and omnivore (O). Codes of species refer to those in ordination diagram. (DOCX) [file pone.0187646.s002.docx]

**S1 Table The species of microfauna of Ladakh soil. Frequency in each elevational transect.** Traits: scraper (S), microbial filtrator (MF), bacterivore (B), fungivore (F), root-fungal feeder (RFF), predator (P), and omnivore (O). Codes of species refer to those in ordination diagram.

|  | Trait | Code | Frequency (%) | |
| --- | --- | --- | --- | --- |
|  |  |  | Zanskar | Tso Moriri |
| Rotifera |  |  |  |  |
| Adineta vaga (Davis, 1873) | S | Adivag | 0 | 13.3 |
| Ceratotrocha cornigera (Bryce, 1893) | MF | Cercor | 20.0 | 6.7 |
| Ceratotrocha sp. 1 | MF | Cersp1 | 6.7 | 13.3 |
| Habrotrocha flaviformis De Koning, 1947 | MF | Habfla | 13.3 | 6.7 |
| Habrotrocha spicula Bryce, 1913 | MF | Habspi | 6.7 | 0 |
| Habrotrocha insignis Bryce, 1915 | MF | Habins | 26.7 | 6.7 |
| Habrotrocha pusila (Bryce, 1893) | MF | Habpus | 6.7 | 0 |
| Habrotrocha sp. 1 | MF | Habsp1 | 13.3 | 6.7 |
| Habrotrocha sp. 2 | MF | Habsp2 | 6.7 | 13.3 |
| Habrotrocha sp. 3 | MF | Habsp3 | 0 | 6.7 |
| Habrotrocha sp. 4 | MF | Habsp4 | 6.7 | 0 |
| Macrotrachela habita (Bryce, 1912) | MF | Machab | 20.0 | 0 |
| Macrotrachela nana (Bryce, 1912) | MF | Macnan | 6.7 | 0 |
| Macrotrachela sp. | MF | Macsp. | 20.0 | 0 |
| Mniobia sp. 1 | MF | Mnisp. | 0 | 13.3 |
| Otostephanos torquatus (Bryce, 1913) | MF | Ototor | 6.7 | 0 |
| Rotaria sp. 1 | MF | Rotsp1 | 6.7 | 0 |
| Rotaria sp. | MF | Rotsp. | 0 | 13.3 |
| Scepanotrocha sp. | MF | Scesp. | 13.3 | 0 |
| Nematoda |  |  |  |  |
| Acrobeles sp. juv. | B | Acrobe | 0 | 13.3 |
| Acrobeloides tricornis (Thorne, 1925) s. l. | B | Acrdes | 26.7 | 13.3 |
| Acromoldavicus cf. mojavicus Baldwin et al. 2001 | B | Acromo | 0 | 26.7 |
| Aphelenchoides sp. 1 | F | Aphde1 | 0 | 6.7 |
| Aphelenchoides sp. 2 | F | Aphde2 | 0 | 20.0 |
| Aphelenchoides sp. 3 | F | Aphde3 | 0 | 13.3 |
| Aphelenchoides sp. 4 cf. obtusus Thorne & Malek, 1968 | F | Aphde4 | 0 | 33.3 |
| Aphelenchoides sp. 5 cf. macromucleatus Baranovskaya, 1963 | F | Aphde5 | 0 | 6.7 |
| Aphelenchoides sp. 6 cf. lagenoferrus Baranovskaya, 1963 | F | Aphde6 | 33.3 | 13.3 |
| Aphelenchoides sp. 7 | F | Aphde7 | 33.3 | 6.7 |
| Aphelenchus avenae Bastian, 1865 juv. | F | Apchus | 6.7 | 0 |
| Chiloplacus sp. juv. | B | Chilpl | 6.7 | 26.7 |
| Cephalobidae sp. (Metacrolobus?) | B | Cepdae | 0 | 6.7 |
| Cervidellus hamatus Thorne, 1937 | B | Cervi1 | 0 | 20.0 |
| Cervidellus neftasiensis Boström. 1986 | B | Cervi2 | 6.7 | 0 |
| Crassolabium sp. 1 | O | Crass1 | 26.7 | 20.0 |
| Crassolabium sp. 2 | O | Crass2 | 0 | 13.3 |
| Ditylenchus sp. | F | Dityle | 6.7 | 0 |
| Dorylaimida juveniles | O | DOjuvs | 20.0 | 13.3 |
| Eucephalobus oxyuroides (de Man, 1876) juv. | B | Euceph | 6.7 | 0 |
| Eudorylaimus sp. 1 cf. altherri Tjepkema, Ferris & Ferris, 1971 | O | Eudor1 | 0 | 20.0 |
| Eudorylaimus sp. 2 (male+juveniles) | O | Eudor2 | 20.0 | 20.0 |
| Eudorylaimus sp. 3 juv. | O | Eudor3 | 0 | 6.7 |
| Filenchus butteus (Thorne & Malek, 1968) | RFF | Filen2 | 0 | 6.7 |
| Filenchus quartus (Szczygieł, 1969) | RFF | Filen1 | 93.3 | 13.3 |
| Heterodorus cf. brevidentatus (Thorne, 1939) | O | Hedor1 | 40.0 | 0 |
| Heterodorus sp. juv. | O | Hedor2 | 20.0 | 0 |
| Mesodorylaimus cf. subtilis (Thorne & Swanger, 1936) group | O | Mesod1 | 0 | 20.0 |
| Nothotylenchus sp. | F | Nothot | 6.7 | 6.7 |
| Panagrolaimus cf. rigidus (Schneider, 1866) | B | Panagr | 46.7 | 40.0 |
| Paraphelenchus pseudoparietinus Micoletzky, 1922 | F | Paraph | 13.3 | 20.0 |
| Paravulvus sp. 1 | P | Parav1 | 0 | 13.3 |
| Paravulvus sp. 2 juv. | P | Parav2 | 0 | 6.7 |
| Plectus acuminatus Bastian, 1865 | B | Plect2 | 6.7 | 0 |
| Plectus sp. (cf. communis Bütschli, 1873 group) | B | Plect1 | 33.3 | 40.0 |
| Rhabdolaimus terrestris de Man, 1880 | B | Rhbdol | 0 | 13.3 |
| Stegelletina devimucronata (Sumenkova, 1964) | B | Stege1 | 6.7 | 13.3 |
| Stegelletina similis (Thorne, 1925) | B | Stege2 | 13.3 | 6.7 |
| Teratocephalus dadayi Andrássy, 1968 | B | Terato | 0 | 6.7 |
| Tylenchus naranensis Maqbool, Zarina & Ghazala, 1987 | RFF | Tylenc | 40.0 | 0 |
| Tardigrada | O | Tardig | 0 | 13.3 |
