# Supplementary material for: Diversity and feeding strategies of soil microfauna along elevation gradients in Himalayan cold deserts
Source: PLoS One. 2017 Nov 13;12(11):e0187646. doi: 10.1371/journal.pone.0187646 (PMC5683576; doi:10.1371/journal.pone.0187646)
Supplement: S2 Table — (PDF) [file pone.0187646.s003.pdf]

		Z1	Z2	Z3	Z4	Z5	T1	T2
Adivaga	mean	0,000	0,000	0,000	0,000	0,000	55,335	53,642
	SD	0,000	0,000	0,000	0,000	0,000	78,256	75,861
Cercorn	mean	0,000	0,000	232,385	0,000	228,740	0,000	50,407
	SD	0,000	0,000	328,642	0,000	239,543	0,000	71,286
Cermont	mean	0,000	0,000	234,056	0,000	0,000	0,000	0,000
	SD	0,000	0,000	331,005	0,000	0,000	0,000	0,000
Habflav	mean	0,000	0,000	0,000	0,000	182,114	0,000	0,000
	SD	0,000	0,000	0,000	0,000	175,738	0,000	0,000
Habspic	mean	0,000	0,000	58,514	0,000	0,000	0,000	0,000
	SD	0,000	0,000	82,751	0,000	0,000	0,000	0,000
Habprox	mean	0,000	137,331	58,514	0,000	233,130	50,491	0,000
	SD	0,000	97,314	82,751	0,000	329,696	71,405	0,000
Habpusi	mean	0,000	0,000	0,000	0,000	139,878	0,000	0,000
	SD	0,000	0,000	0,000	0,000	197,817	0,000	0,000
Habsp.1	mean	0,000	0,000	135,976	0,000	0,000	0,000	50,407
	SD	0,000	0,000	98,910	0,000	0,000	0,000	71,286
Habsp.2	mean	0,000	0,000	175,542	0,000	0,000	0,000	0,000
	SD	0,000	0,000	248,254	0,000	0,000	0,000	0,000
Habsp.3	mean	0,000	0,000	0,000	0,000	0,000	0,000	53,642
	SD	0,000	0,000	0,000	0,000	0,000	0,000	75,861
Habsp.4	mean	0,000	0,000	73,464	0,000	0,000	0,000	0,000
	SD	0,000	0,000	103,894	0,000	0,000	0,000	0,000
Machabi	mean	0,000	0,000	175,542	57,633	139,878	0,000	0,000
	SD	0,000	0,000	248,254	81,506	197,817	0,000	0,000
Macnana	mean	0,000	0,000	58,514	0,000	0,000	0,000	0,000
	SD	0,000	0,000	82,751	0,000	0,000	0,000	0,000
Macsp.	mean	69,151	0,000	0,000	115,266	46,626	0,000	0,000
	SD	97,794	0,000	0,000	163,011	65,939	0,000	0,000
Mnisp.	mean	0,000	0,000	0,000	0,000	0,000	0,000	0,000
	SD	0,000	0,000	0,000	0,000	0,000	0,000	0,000
Ototorq	mean	0,000	0,000	220,393	0,000	0,000	0,000	0,000
	SD	0,000	0,000	311,683	0,000	0,000	0,000	0,000
Rotsp.1	mean	0,000	72,290	0,000	0,000	0,000	0,000	0,000
	SD	0,000	102,233	0,000	0,000	0,000	0,000	0,000
Rotsp.2	mean	0,000	0,000	0,000	0,000	0,000	0,000	151,220
	SD	0,000	0,000	0,000	0,000	0,000	0,000	213,857
Scesp.	mean	0,000	66,079	440,786	0,000	0,000	0,000	0,000
	SD	0,000	93,451	623,365	0,000	0,000	0,000	0,000
Tardigr	mean	0,000	0,000	0,000	0,000	0,000	150,508	0,000
	SD	0,000	0,000	0,000	0,000	0,000	123,685	0,000
Rhbdol	mean	0,000	0,162	0,000	0,000	0,000	0,000	0,000
	SD	0,000	0,133	0,000	0,000	0,000	0,000	0,000
Plect.1	mean	0,000	0,943	0,849	0,074	0,000	0,055	0,000
	SD	0,000	0,368	0,726	0,104	0,000	0,077	0,000
Plect.2	mean	0,000	0,000	0,000	0,000	0,000	0,055	0,000
	SD	0,000	0,000	0,000	0,000	0,000	0,077	0,000
Terato	mean	0,000	0,053	0,000	0,000	0,000	0,000	0,000
	SD	0,000	0,075	0,000	0,000	0,000	0,000	0,000
Euceph	mean	0,000	0,000	0,000	0,000	0,000	0,000	0,052

	SD	0,000	0,000	0,000	0,000	0,000	0,000	0,074
Acrdes	mean	0,000	0,000	0,148	0,147	0,000	0,000	0,000
	SD	0,000	0,000	0,209	0,208	0,000	0,000	0,000
Acromo	mean	0,000	0,000	0,699	0,566	0,000	0,000	0,000
	SD	0,000	0,000	0,989	0,141	0,000	0,000	0,000
Chilpl	mean	0,000	0,051	0,049	0,000	0,105	0,000	0,000
	SD	0,000	0,072	0,070	0,000	0,074	0,000	0,000
Cervi.1	mean	0,000	0,000	0,152	0,000	0,055	0,000	0,000
	SD	0,000	0,000	0,111	0,000	0,078	0,000	0,000
Cerv.i2	mean	0,000	0,000	0,000	0,000	0,000	0,000	0,052
	SD	0,000	0,000	0,000	0,000	0,000	0,000	0,074
Stege.1	mean	0,000	0,000	0,000	0,282	0,000	0,000	0,000
	SD	0,000	0,000	0,000	0,279	0,000	0,000	0,000
Stege.2	mean	0,000	0,000	0,000	0,061	0,000	0,000	0,000
	SD	0,000	0,000	0,000	0,086	0,000	0,000	0,000
Acrobe	mean	0,000	0,000	0,087	0,074	0,000	0,000	0,000
	SD	0,000	0,000	0,124	0,104	0,000	0,000	0,000
Cepdae	mean	0,000	0,000	0,087	0,000	0,000	0,000	0,000
	SD	0,000	0,000	0,124	0,000	0,000	0,000	0,000
Panagr	mean	0,000	0,462	27,266	0,000	0,152	0,054	0,000
	SD	0,000	0,448	34,022	0,000	0,125	0,077	0,000
Apchus	mean	0,000	0,000	0,000	0,000	0,000	0,000	0,000
	SD	0,000	0,000	0,000	0,000	0,000	0,000	0,000
Paraph	mean	0,000	0,000	1,409	0,244	0,000	0,000	0,000
	SD	0,000	0,000	1,184	0,345	0,000	0,000	0,000
Aphde.1	mean	0,000	0,158	0,000	0,000	0,000	0,000	0,000
	SD	0,000	0,224	0,000	0,000	0,000	0,000	0,000
Aphd.e2	mean	0,000	0,156	0,049	0,000	0,000	0,000	0,000
	SD	0,000	0,125	0,070	0,000	0,000	0,000	0,000
Aphde.3	mean	0,000	0,102	0,099	0,000	0,000	0,000	0,000
	SD	0,000	0,144	0,139	0,000	0,000	0,000	0,000
Aphde.4	mean	0,000	0,000	8,741	6,539	0,051	0,000	0,000
	SD	0,000	0,000	12,257	7,748	0,072	0,000	0,000
Aphde.5	mean	0,000	0,000	0,193	0,000	0,000	0,000	0,000
	SD	0,000	0,000	0,273	0,000	0,000	0,000	0,000
Aphde.6	mean	0,000	0,053	0,690	0,000	0,000	0,054	0,000
	SD	0,000	0,075	0,975	0,000	0,000	0,077	0,000
Aphde.7	mean	0,000	0,000	0,000	0,074	0,000	0,272	0,000
	SD	0,000	0,000	0,000	0,104	0,000	0,204	0,000
Nothot	mean	0,000	0,000	0,000	0,122	0,000	0,000	0,000
	SD	0,000	0,000	0,000	0,172	0,000	0,000	0,000
Dityle	mean	0,000	0,000	0,000	0,000	0,000	0,000	0,000
	SD	0,000	0,000	0,000	0,000	0,000	0,000	0,000
Filen.1	mean	0,000	0,209	0,000	0,000	0,000	0,218	1,589
	SD	0,000	0,198	0,000	0,000	0,000	0,204	0,768
Filen.2	mean	0,000	0,000	0,345	0,000	0,000	0,000	0,000
	SD	0,000	0,000	0,488	0,000	0,000	0,000	0,000
Tylenc	mean	0,000	0,000	0,000	0,000	0,000	0,273	0,156
	SD	0,000	0,000	0,000	0,000	0,000	0,278	0,221
DOjuvs	mean	0,000	0,102	0,193	0,000	0,000	0,000	0,000

	SD	0,000	0,144	0,273	0,000	0,000	0,000	0,000
Mesod.1	mean	0,340	0,000	0,000	0,000	0,000	0,000	0,000
	SD	0,143	0,000	0,000	0,000	0,000	0,000	0,000
Eudor.1	mean	0,180	0,102	0,000	0,000	0,000	0,000	0,000
	SD	0,153	0,144	0,000	0,000	0,000	0,000	0,000
Eudor.2	mean	0,000	0,000	0,087	0,269	0,000	0,000	0,000
	SD	0,000	0,000	0,124	0,193	0,000	0,000	0,000
Eudor.3	mean	0,000	0,053	0,000	0,000	0,000	0,000	0,000
	SD	0,000	0,075	0,000	0,000	0,000	0,000	0,000
Crass.2	mean	0,000	0,160	0,000	0,000	0,000	0,000	0,000
	SD	0,000	0,133	0,000	0,000	0,000	0,000	0,000
Crass.1	mean	0,000	0,102	0,216	0,000	0,000	0,000	0,062
	SD	0,000	0,144	0,161	0,000	0,000	0,000	0,088
Hedor.1	mean	0,000	0,000	0,000	0,000	0,000	0,218	0,000
	SD	0,000	0,000	0,000	0,000	0,000	0,154	0,000
Hedor.2	mean	0,000	0,000	0,000	0,000	0,000	0,055	0,062
	SD	0,000	0,000	0,000	0,000	0,000	0,077	0,088
Parav.2	mean	0,000	0,053	0,000	0,000	0,000	0,000	0,000
	SD	0,000	0,075	0,000	0,000	0,000	0,000	0,000
Parav.1	mean	0,000	0,160	0,000	0,000	0,000	0,000	0,000
	SD	0,000	0,129	0,000	0,000	0,000	0,000	0,000
FF	mean	311,179	421,319	1859,146	868,202	1330,203	50,491	414,651
	SD	311,179	354,582	1227,162	618,491	1456,364	71,405	249,681
T	mean	0,000	0,000	0,000	0,000	0,000	150,508	0,000
	SD	0,000	0,000	0,000	0,000	0,000	123,685	0,000
BV	mean	0,123	0,319	2,022	0,451	3,375	0,720	1,532
	SD	0,123	0,226	2,678	0,469	2,811	1,018	1,085
FV	mean	0,736	0,000	0,760	0,183	1,124	1,944	0,521
	SD	0,001	0,000	1,075	0,259	0,834	2,749	0,541
RF	mean	0,614	2,600	3,064	4,388	1,276	5,616	0,317
	SD	0,614	1,177	3,356	3,899	0,800	7,942	0,448
OV	mean	0,368	0,334	0,087	0,085	2,116	0,441	0,741
	SD	0,368	0,237	0,123	0,120	1,375	0,127	0,418
P	mean	0,000	0,000	0,000	0,000	0,000	0,000	0,416
	SD	0,000	0,000	0,000	0,000	0,000	0,000	0,397
AT	mean	3806,000	4078,333	4458,000	4638,667	4480,000	5579,000	5941,333
	SD	1,000	194,738	142,836	63,379	165,664	842,165	166,534
MS	mean	26,216	22,492	30,759	39,392	33,218	15,781	13,683
	SD	0,000	6,861	16,456	14,771	11,104	7,850	2,248
OM	mean	7,088	9,743	11,313	10,205	7,624	2,607	1,498
	SD	0,000	2,516	6,322	2,388	2,557	2,755	0,978
NH4	mean	13,635	7,506	5,047	5,750	2,761	1,091	0,686
	SD	0,000	4,713	2,367	2,152	0,636	0,607	0,075
NO3	mean	1,802	2,034	45,099	39,751	43,943	7,531	3,203
	SD	0,000	0,490	44,172	16,020	25,135	8,611	1,977
TN	mean	2708,327	3655,265	7646,506	3652,563	2801,058	947,309	756,255
	SD	0,000	752,782	6639,390	659,142	990,104	990,658	597,537
PO4	mean	26,808	24,469	34,343	17,168	30,233	18,755	9,148
	SD	0,000	2,188	5,649	5,096	9,836	19,969	4,014
Na	mean	0,256	0,229	0,257	0,221	0,219	0,184	0,175

	SD	0,000	0,019	0,007	0,030	0,003	0,025	0,020
K	mean	2,200	1,995	4,302	4,066	2,245	1,895	1,786
	SD	0,000	0,174	0,481	2,000	0,406	0,717	0,081
Ca	mean	0,586	0,737	0,602	0,261	0,164	0,941	0,490
	SD	0,000	0,129	0,201	0,190	0,102	0,461	0,049
Mg	mean	10,922	12,483	8,231	6,981	2,368	2,526	2,525
	SD	0,000	1,116	0,433	3,361	0,666	1,845	0,468
pH	mean	6,100	5,583	5,137	4,880	6,220	7,923	6,787
	SD	0,000	0,369	0,737	1,020	1,216	0,486	0,547

T3	T4	T5
0,000	0,000	0,000
0,000	0,000	0,000
0,000	0,000	0,000
0,000	0,000	0,000
100,136	55,827	0,000
141,613	78,951	0,000
801,084	0,000	0,000
1132,904	0,000	0,000
0,000	0,000	0,000
0,000	0,000	0,000
0,000	0,000	0,000
0,000	0,000	0,000
0,000	0,000	0,000
0,000	0,000	0,000
0,000	0,000	0,000
0,000	0,000	0,000
255,488	0,000	0,000
262,371	0,000	0,000
0,000	0,000	0,000
0,000	0,000	0,000
0,000	0,000	0,000
0,000	0,000	0,000
0,000	0,000	0,000
0,000	0,000	0,000
0,000	0,000	0,000
0,000	0,000	0,000
0,000	0,000	0,000
0,000	0,000	0,000
50,068	52,337	0,000
70,806	74,016	0,000
0,000	0,000	0,000
0,000	0,000	0,000
0,000	0,000	0,000
0,000	0,000	0,000
154,065	0,000	0,000
217,881	0,000	0,000
0,000	0,000	0,000
0,000	0,000	0,000
0,000	0,000	0,000
0,000	0,000	0,000
0,000	0,000	0,000
0,000	0,000	0,000
0,074	0,000	0,623
0,345	0,000	0,178
0,000	0,000	0,000
0,000	0,000	0,000
0,000	0,000	0,000
0,000	0,000	0,000
0,000	0,000	0,000

0,000	0,000	0,000
0,147	0,000	0,221
0,068	0,000	0,146
0,566	0,000	0,000
0,000	0,000	0,000
0,000	0,000	0,037
0,000	0,000	0,053
0,000	0,000	0,000
0,000	0,000	0,000
0,000	0,000	0,000
0,000	0,000	0,000
0,282	0,000	0,000
0,068	0,000	0,000
0,061	0,000	0,095
0,203	0,000	0,134
0,074	0,000	0,000
0,000	0,000	0,000
0,000	0,000	0,000
0,000	0,000	0,000
0,000	0,057	1,124
1,517	0,080	0,827
0,000	0,000	0,048
0,000	0,000	0,067
0,244	0,000	0,571
0,000	0,000	0,564
0,000	0,000	0,000
0,000	0,000	0,000
0,000	0,000	0,000
0,000	0,000	0,000
0,000	0,000	0,000
0,000	0,000	0,000
6,539	0,000	0,000
0,000	0,000	0,000
0,000	0,000	0,000
0,000	0,000	0,000
0,000	0,000	0,282
0,479	0,000	0,258
0,074	0,000	0,637
0,135	0,000	0,549
0,122	0,000	0,000
0,068	0,000	0,000
0,000	0,000	0,037
0,000	0,000	0,053
0,000	0,780	3,287
2,636	0,092	3,776
0,000	0,000	0,000
0,000	0,000	0,000
0,000	0,314	0,037
0,000	0,225	0,053
0,000	0,000	0,483

0,000	0,000	0,265
0,000	0,000	0,000
0,000	0,000	0,000
0,000	0,000	0,000
0,000	0,000	0,000
0,269	0,000	0,168
0,000	0,000	0,059
0,000	0,000	0,000
0,000	0,000	0,000
0,000	0,000	0,000
0,000	0,000	0,000
0,000	0,000	0,178
0,082	0,000	0,127
0,000	0,129	0,184
0,000	0,093	0,180
0,000	0,000	0,095
0,000	0,000	0,134
0,000	0,000	0,000
0,000	0,000	0,000
0,000	0,000	0,000
0,000	0,000	0,000
868,202	466,667	52,337
1346,101	545,840	74,016
0,000	0,000	0,000
0,000	0,000	0,000
0,451	5,271	0,814
71,107	4,760	0,299
0,183	24,341	1,956
3,510	19,008	2,555
4,388	0,000	0,000
0,912	0,000	0,000
0,085	0,727	0,206
0,896	0,620	0,291
0,000	0,000	0,000
0,000	0,000	0,000
4638,667	5151,667	4655,333
172,380	194,172	243,765
39,392	5,734	1,274
15,323	2,973	0,685
10,205	1,474	0,633
1,027	0,495	0,113
5,750	2,015	2,364
0,358	0,431	2,178
39,751	3,314	1,042
11,737	1,511	0,920
3652,563	669,053	265,860
95,788	93,234	105,605
17,168	42,496	32,455
7,071	5,659	12,382
0,221	0,139	0,266

0,020	0,017	0,089
4,066	1,801	1,139
1,158	0,316	0,235
0,261	0,394	2,336
0,271	0,059	1,592
6,981	2,817	2,382
2,207	0,620	0,178
4,880	6,897	8,050
0,743	0,522	0,635
